# A novel model for photothermal excitation of variable thermal conductivity semiconductor elastic medium subjected to mechanical ramp type with two-temperature theory and magnetic field

**DOI:** 10.1038/s41598-019-39955-z

**Published:** 2019-03-01

**Authors:** Kh. Lotfy

**Affiliations:** Math. Department, Faculty of Science, Zagazig University, Zagazig, 44519 Egypt

## Abstract

This article focuses on studying the dependence of the thermal conductivity of a semiconducting medium on temperature in context of photothermal transport process and variable thermal conductivity, chosen to be linear function in temperature. The effect of the initial magnetic field is introduced in the problem governing equations with the two-temperature theory. The complete solution in one dimension is obtained using Laplace transform technique. The thermal heating ramp type and mechanical load throughout the elastic-photothermal excitation are considered in the problem boundary conditions. Some physical fields are obtained by using numerical inversion of the Laplace transform and Riemann-sum approximation method. The thermo-dynamical temperature, conductive temperature, displacement, strain, normal stress and carrier density are discussed and shown graphically with some comparisons.

## Introduction

In material science the variable thermal conductivity which depends on temperature is very important and has many applications in the nature. The recent studies of semiconductors thermal conductivity dependence on temperature showed that, the physical properties especial the deformation and the thermo-mechanical behavior are strongly effected by any changes in the materiel temperature. Thermal conductivity of materials such as silicon nitride reduced by half for temperatures between 1.0 °C and 400.0 °C. In general, semiconductor materials are very sensitive with temperature changes. When the semiconductor surface is exposed to weak beam of laser, free carriers (free charge) are generated. When studying thermal stress problem, the dependence of the material on the temperature should be taken in consideration especially when the thermal conductivity of the material is under investigation.

In the early years of the last century, Suhara studied the effect of temperature on a hollow cylinder. Many elastic and inelastic media problems take into consideration the dependence of the physical properties on temperature^1^. Youssef^2^ used the state-space approach to solve the generalized thermoelasticity problem for elastic medium with spherical cavity when the thermal conductivity depends on temperature with ramp-type heating. Also variable thermal conductivity is applied on the generalized thermoelasticity layered with thermal shock by Youssef and El-Bary^3^. The solution of the generalized thermoelasticity problem with variable thermal conductivity for infinite long annular cylinder is discussed by Youssef and Abbas^4^. Many applications in oil extraction that depends on variation of pressure and temperature are taken from the study of the generalized thermoelasticity.

When a beam of laser falls on the external surface of semiconductor material, the excited electrons generate the carrier free charge intensity and plasma waves. That is the main reason for the governing equations of the thermoelasticity problems observe the thermal-elastic waves. Most of the previous studies ignored the interaction between thermal, elastic and plasma waves. Recently the interplay between these waves is confirmed experimentally and theoretically. The electronic deformation mechanism is studied by Photoacoustic frequency transmission technique^5^. The photoacoustic spectroscopy analysis is used to obtain the photothermal method when a beam falls from a laser on a semiconductor elastic material^6,7^. The photothermal method is used to determine the values of some physical variables of certain materials^5,8–11^. Opsal and Rosencwaig^12^ studied the plasma -thermal-elastic waves are in elastic silicon. Song *et al*.^13^ used the optically excited semiconductor to study the vibration of thermoelastic medium. Lotfy *et al*.^14–19^ used the thermal memory to discuss the overlap between the Photothermal and the generalized thermoelasticty theories in semiconductor materials with different external fields. Hobiny and Abbas^20,21^ studied the non-homogenous semiconductor materials with cylindrical cavity by Photothermal theory. Abo-Dahab and Lotfy^22^ used the two-temperature theory to explain the photo-excitation of a semiconductor medium.

The first to know the theory of two temperatures were Chen and Gurtin^23^ and Chen *et al*.^24^. They have corrected a heat conduction theory for deformable bodies, which be based on two different distinct temperatures, which called the thermodynamic temperature *T* and the conductive temperature *φ*. In cases of time independent, the variation between these two temperatures is proportional to supply of heat, when the heat supply is absence the two temperatures are equal. However, especially in case of the problems of wave propagation, the two temperatures are in general different, regardless of the presence of a heat supply^25^. Youssef^26^ used the model of two temperature theory in generalized thermoelasticity which show that the two temperature theory can differentiates between the wave propagation of the temperature, that comes from heat conduction temperature (thermal process) and that which comes from the thermodynamics temperature (mechanical process).

In this article we study the dependence of the variable thermal conductivity on temperature. Based on the material properties, the variable thermal conductivity is taken linear function in temperature. More attention is given to study the interaction between elastic-thermal-plasma waves through Photothermal process under the effect of magnetic field. The one dimensional two-temperature theory is used to determine the problem state variables of the semiconductor medium subjected to mechanical ramp type and thermal shock problem. According to the Riemann-sum approximation method, the Laplace transform and the inversion Laplace method are used to transform the governing equations and obtaining the results. The solutions are shown graphically with discussions.

## Basic Equations

Considering a semiconductor elastic medium under the influence of constant initial magnetic field, $\mathop{H}\limits^{\rightharpoonup }=(0,{H}_{0},0)$. The Maxwell’s equations with linearized electromagnetism slowly moving medium are:

1 $$\mathop{J}\limits^{\rightharpoonup }=curl\,\mathop{h}\limits^{\rightharpoonup },$$

2 $$curl\,\overrightarrow{E}=-\,{\mu }_{0}\mathop{\dot{H}}\limits^{\rightharpoonup },$$

3 $$\mathop{E}\limits^{\rightharpoonup }=-\,{\mu }_{0}(\mathop{\dot{u}}\limits^{\rightharpoonup }\times \,\mathop{H}\limits^{\rightharpoonup }),$$

4 $$div\,\mathop{h}\limits^{\rightharpoonup }=0,$$

where, *μ*_0_ is the vacuum magnetic permeability and $\mathop{\dot{u}}\limits^{\rightharpoonup }$ is the particle velocity in the medium (the dote denotes differentiation with respect to time). The current density $\mathop{J}\limits^{\rightharpoonup }$ and the electric field $\mathop{E}\limits^{\rightharpoonup }$ have the same the direction.

Assume that, the induced magnetic field $\mathop{h}\limits^{\rightharpoonup }$ in *y*-direction and the induced electric field $\mathop{E}\limits^{\rightharpoonup }$ in *z*-direction, i. e., $\mathop{h}\limits^{\rightharpoonup }=(0,h,0)$ and $\mathop{E}\limits^{\rightharpoonup }=(0,0,E)$. Also define the displacement vector as $\overrightarrow{u}=(u,0,0)$, and the strain tensor as $e={u}_{x}=\frac{\partial u}{\partial x}$, *u* is a function of *x* and *t* only.

Hence, the only one component current density for slowly moving medium is:

5 $${J}_{z}=\frac{\partial \,h}{\partial \,x}.$$

The electric and magnetic field components expressed in terms of displacement component are:

6 $${E}_{z}=-\,{\mu }_{0}\,{H}_{0}\,\dot{u},$$

7 $$h=-\,{H}_{0}\,e.$$

When a beam of laser with energy *E* incident on a semiconductor elastic surface with gap energy *E*_*g*_ (*E* > *E*_*g*_). The optical energy absorbed in an elastic semiconductor medium causes a change in the thermo-elastic and electronic deformation. These deformations are the mechanisms used to control the curvature of the microstructure. The photo-generated (heat) plasma can produce elastic vibrations (local strain), these vibrations cause the deformation in the semiconductor elastic sample^27^. The general equations of the coupled thermo-elastic waves and plasma waves depend on three principal variable *N* ($\overrightarrow{r}$, t), *T* ($\overrightarrow{r}$, t) and $\mathop{u}\limits^{\rightharpoonup }$ ($\overrightarrow{r}$, t), where *N*($\overrightarrow{r}$, t) is the carrier density (plasma wave), *T* ($\overrightarrow{r}$, t) is the temperature distribution (thermal wave), and $\mathop{u}\limits^{\rightharpoonup }$ ($\overrightarrow{r}$, t) is the displacement distribution (elastic wave), $\overrightarrow{r}$ is the position vector.

Through the excitation transient process, the coupled plasma-thermal-elastic transport equations in two-temperature field take the general (vector form in the time-space coordinates) form^28^:

(I) The plasma and thermal distribution coupled equation takes the form:
  8 $$\frac{\partial N(\mathop{r}\limits^{\rightharpoonup },t)}{\partial t}={D}_{E}{\nabla }^{2}N(\mathop{r}\limits^{\rightharpoonup },t)-\frac{N(\mathop{r}\limits^{\rightharpoonup },t)}{\tau }+\kappa \,T(\mathop{r}\limits^{\rightharpoonup },t).$$
(II) The equation of motion, which describes the relation between the elastic-thermal distribution and the magnetic field is:
  9 $$\rho \frac{{\partial }^{2}\overrightarrow{u}(\mathop{r}\limits^{\rightharpoonup },t)}{\partial {t}^{2}}=\mu {\nabla }^{2}\overrightarrow{u}(\mathop{r}\limits^{\rightharpoonup },t)+(\mu +\lambda )\,\nabla \,(\nabla .\overrightarrow{u}(\mathop{r}\limits^{\rightharpoonup },t))-\gamma \nabla {\rm{T}}(\mathop{r}\limits^{\rightharpoonup },t)-{\delta }_{n}\nabla N(\mathop{r}\limits^{\rightharpoonup },t)+{\mu }_{0}(\mathop{J}\limits^{\rightharpoonup }\times \mathop{H}\limits^{\rightharpoonup }),$$
(III) The coupled equation between plasma and thermo-dynamical distribution in two-temperature theory (energy balance equation) is:
  10 $$\frac{K}{k}\frac{\partial {\rm{T}}(\mathop{r}\limits^{\rightharpoonup },t)}{\partial t}=\nabla .(K\nabla \phi (\mathop{r}\limits^{\rightharpoonup },t))-\frac{{E}_{g}}{\tau }N(\mathop{r}\limits^{\rightharpoonup },t)+\gamma {T}_{0}\nabla .\frac{\partial \overrightarrow{u}(\mathop{r}\limits^{\rightharpoonup },t)}{\partial t}.$$
  where $\kappa =\frac{\partial q}{\partial T}\frac{T}{\tau }$ is the thermal activation coupling parameter and at temperature *T* the concentration of the equilibrium carrier is defined as *q*^29,30^, *D*_*E*_ and *τ* are the diffusion coefficient of the carrier and photogenerated carrier lifetime respectively. The energy gap of the material is *E*_*g*_ (the term $\frac{{E}_{g}}{\tau }N$ represent the influence of heat production by recombining the carriers free charge in the semiconductor sample volume). The constants *μ* and *λ* are Lame’s constants of, *ρ* is the density and the absolute thermo-dynamic temperature is *T*_0_, *γ* is the volume thermal expansion, *α*_*T*_ (*γ* = (3*λ* + 2 *μ*)*α*_*T*_) is the coefficient of the linear thermal expansion, the specific heat of the elastic material is *C*_*e*_, *δ*_*n*_ = (2 *μ* + 3*λ*)*d*_*n*_ is the deformation potential difference, $\frac{K}{\rho {C}_{e}}=k$ is the diffusivity, *K* represents the thermal conductivity in general case and *d*_*n*_ is the coefficient of electronic deformation. In the present study we consider the general case in which thermal activation coupling parameter is non-zero and the medium is linear with homogeneous properties.
(IV) The equation of the heat conduction and the dynamical heat (thermo-dynamical) in two temperatures theory takes the form
  11 $$T=\phi -a{\nabla }^{2}\phi ,$$
  where *a* > 0 is the two-temperature parameter describes temperature discrepancy
(V) The stress–strain–temperature-plasma equation in 1D represent is:

12 $$\sigma ={{\rm{\sigma }}}_{{\rm{xx}}}=(2\mu +\lambda )\frac{\partial u}{\partial x}+(3\lambda +2\mu )({\alpha }_{T}\,T+{d}_{n}N).$$

In semiconductor material, the thermal conductivity parameter is the important one because it determines the thermoelectric energy conversion. The heat conduction affect on electrical resistivity of semiconductors^31,32^.

Considering the thermal conductivity of the semiconductor elastic material *K* can be expressed in a linear form of thermo-dynamical temperature as:

13 $$K(T)={K}_{0}(1+{K}_{1}T),$$

where *K*_0_ is a constant which, in the case of the material, is independent of temperature and equal to the thermal conductivity (*K*_1_ = 0). *K*_1_ is a small non-positive parameter.

From Eqs (11) and (13) we can write:

$$K(T)={K}_{0}(1+{K}_{1}(\phi -a{\nabla }^{2}\phi )).$$

Neglecting the value of *K*_1_*a*∇^2^*φ* (very small value) we can write:

14 $$K(T)={K}_{0}(1+{K}_{1}\phi ).$$

or

15 $$K(\phi )={K}_{0}(1+{K}_{1}\phi ).$$

Introduce the mappings^33^:

16 $$\hat{\phi }=\frac{1}{{K}_{0}}{\int }_{0}^{\phi }K({\rm{\Omega }})d{\rm{\Omega }},$$

17 $$\hat{T}=\frac{1}{{K}_{0}}{\int }_{0}^{T}K({\rm{\Omega }})d{\rm{\Omega }},$$

Operating by $\frac{\partial }{\partial {x}_{i}}$ on the both sides of Eq. (16), yields

18 $${K}_{0}\frac{\partial \hat{\phi }}{\partial {x}_{i}}=K(\phi )\frac{\partial \phi }{\partial {x}_{i}}\,\mathop{\Leftarrow \Rightarrow }\limits^{or\,in\,tensor\,form}\,{K}_{0}{\hat{\phi }}_{,i}=K(\phi ){\phi }_{,i}.$$

Again, differentiating Eqs (17) and (18) with respect to *x*_*i*_:

19 $${K}_{0}\frac{{\partial }^{2}\hat{\phi }}{\partial {x}_{i}^{2}}=\frac{\partial }{\partial {x}_{i}}(K(\phi )\frac{\partial \phi }{\partial {x}_{i}})\,\mathop{\Leftarrow \Rightarrow }\limits^{or\,in\,tensor\,form}\,{K}_{0}{\hat{T}}_{,i}=K(T){T}_{,i}.$$

20 $${K}_{0}\frac{\partial \hat{T}}{\partial {x}_{i}}=K(T)\frac{\partial T}{\partial {x}_{i}}\,\mathop{\Leftarrow \Rightarrow }\limits^{or\,in\,tensor\,form}\,{K}_{0}{\hat{T}}_{,i}=K(T){T}_{,i}.$$

On the other hand, differentiating Eq. (17) with respect to time we obtain

21 $${K}_{0}\frac{\partial \hat{T}}{\partial t}=K(T)\frac{\partial T}{\partial t}.$$

Using Eqs (19), (20) and (21) into Eqs (9) and (10) we obtain:

22 $$\rho \frac{{\partial }^{2}\overrightarrow{u}}{\partial {t}^{2}}=\mu {\nabla }^{2}\overrightarrow{u}+(\mu +\lambda )\,\nabla \,(\nabla .\overrightarrow{u})-\frac{\gamma {K}_{0}}{K(T)}\frac{\partial \hat{T}}{\partial {x}_{i}}-{\delta }_{n}\nabla N+{\mu }_{0}(\mathop{J}\limits^{\rightharpoonup }\times \mathop{H}\limits^{\rightharpoonup }),$$

23 $$\frac{1}{k}\frac{\partial \hat{{\rm{T}}}}{\partial t}={\hat{\phi }}_{,ii}+\frac{{E}_{g}}{{K}_{0}\tau }N(\mathop{r}\limits^{\rightharpoonup },t)+\frac{\gamma {T}_{0}}{{K}_{0}}\nabla .\frac{\partial \overrightarrow{u}}{\partial t}.$$

Using the mappings Eqs (16) and (17) into the two-temperature Eq. (11), after working the following steps:

1. Influencing on Eq. (11) by the operator $\frac{\partial }{\partial {x}_{i}}$ and multiplying both sides by *K* we get:
  24 $$K(T)\frac{\partial T}{\partial {x}_{i}}=K(\phi )\frac{\partial \phi }{\partial {x}_{i}}-aK(\phi )\frac{\partial }{\partial {x}_{i}}\frac{{\partial }^{2}\phi }{\partial {x}_{m}^{2}},\,\,i,m=1,2,3,$$
2. Using Eqs (18) and (20) into Eq. (24), we obtain
  25 $${K}_{0}\frac{\partial \hat{T}}{\partial {x}_{i}}={K}_{0}\frac{\partial \hat{\phi }}{\partial {x}_{i}}-aK(\phi )\frac{\partial }{\partial {x}_{i}}\frac{{\partial }^{2}\phi }{\partial {x}_{m}^{2}},\,\,i,m=1,2,3.$$
3. Eq. (19) can be rewritten in the form:

26 $${K}_{0}\frac{{\partial }^{2}\hat{\phi }}{\partial {x}_{m}^{2}}=\frac{\partial }{\partial {x}_{m}}(K(\phi )\frac{\partial \phi }{\partial {x}_{m}})=\frac{\partial }{\partial {x}_{m}}({K}_{0}(1+{K}_{1}\phi )\frac{\partial \phi }{\partial {x}_{m}}).$$

Differentiating both sides of the above equation with respect to *x*_*i*_, gives

27 $${K}_{0}\frac{\partial }{\partial {x}_{i}}\frac{{\partial }^{2}\hat{\phi }}{\partial {x}_{m}^{2}}=\frac{\partial }{\partial {x}_{i}}[\frac{\partial }{\partial {x}_{m}}({K}_{0}(1+{K}_{1}\phi )\frac{\partial \phi }{\partial {x}_{m}})]=3{K}_{0}{K}_{1}\frac{\partial \phi }{\partial {x}_{i}}\frac{{\partial }^{2}\phi }{\partial {x}_{m}^{2}}+K(\phi )\frac{\partial }{\partial {x}_{i}}\frac{{\partial }^{2}\phi }{\partial {x}_{m}^{2}}.$$

Neglecting the non-linear term of Eq. (27) we get:

28 $$K(\phi )\frac{\partial }{\partial {x}_{i}}\frac{{\partial }^{2}\phi }{\partial {x}_{m}^{2}}={K}_{0}\frac{\partial }{\partial {x}_{i}}\frac{{\partial }^{2}\hat{\phi }}{\partial {x}_{m}^{2}}\,\mathop{\Leftarrow \Rightarrow }\limits^{or\,in\,tensor\,form}\,{K}_{0}{\hat{\phi }}_{,mmi}=K(\phi ){\phi }_{,mmi}.$$

Then Eq. (25) becomes

29 $$\frac{\partial \hat{T}}{\partial {x}_{i}}=\frac{\partial \hat{\phi }}{\partial {x}_{i}}-a\frac{\partial }{\partial {x}_{i}}\frac{{\partial }^{2}\hat{\phi }}{\partial {x}_{m}^{2}}\,\mathop{\Leftarrow \Rightarrow }\limits^{or\,in\,tensor\,form}\,{\hat{T}}_{,i}={\hat{\phi }}_{,i}-a{\hat{\phi }}_{,mmi}.$$

Integrating the above equation,

30 $$\hat{T}=\hat{\phi }-a\frac{{\partial }^{2}\hat{\phi }}{\partial {x}_{m}^{2}}\,\mathop{\Leftarrow \Rightarrow }\limits^{or\,in\,tensor\,form}\,\hat{T}=\hat{\phi }-a{\hat{\phi }}_{,mm}.$$

Similarly, differentiating Eq. (8) with respect to *x*_*i*_, and using (20), we get

31 $$\frac{\partial }{\partial t}\frac{\partial N}{\partial {x}_{i}}={D}_{E}\frac{\partial }{\partial {x}_{i}}\frac{{\partial }^{2}N}{\partial {x}_{m}^{2}}-\frac{1}{\tau }\frac{\partial N}{\partial {x}_{i}}+\kappa \frac{\partial T}{\partial {x}_{i}},$$

32 $$\frac{\partial }{\partial t}\frac{\partial N}{\partial {x}_{i}}={D}_{E}\frac{\partial }{\partial {x}_{i}}\frac{{\partial }^{2}N}{\partial {x}_{m}^{2}}-\frac{1}{\tau }\frac{\partial N}{\partial {x}_{i}}+\frac{\kappa {K}_{0}}{K}\frac{\partial \hat{T}}{\partial {x}_{i}}.$$

Neglecting the non-linear terms, we can write:

33 $$\begin{array}{rcl}\frac{\kappa {K}_{0}}{{K}_{0}(1+{K}_{1}T)}\frac{\partial \hat{T}}{\partial {x}_{i}} & = & \kappa {(1+{K}_{1}T)}^{-1}\frac{\partial \hat{T}}{\partial {x}_{i}}=\kappa (1-{K}_{1}T+{({K}_{1}T)}^{2}-\ldots \ldots .)\frac{\partial \hat{T}}{\partial {x}_{i}}\\  & = & \kappa \frac{\partial \hat{T}}{\partial {x}_{i}}-\kappa {K}_{1}T\frac{\partial \hat{T}}{\partial {x}_{i}}+\kappa {({K}_{1}T)}^{2}\frac{\partial \hat{T}}{\partial {x}_{i}}-\ldots =\kappa \frac{\partial \hat{T}}{\partial {x}_{i}}.\end{array}$$

Substituting from Eq. (33) into Eq. (32), we get

34 $$\frac{\partial }{\partial t}\frac{\partial N}{\partial {x}_{i}}={D}_{E}\frac{\partial }{\partial {x}_{i}}\frac{{\partial }^{2}N}{\partial {x}_{m}^{2}}-\frac{1}{\tau }\frac{\partial N}{\partial {x}_{i}}+\kappa \frac{\partial \hat{T}}{\partial {x}_{i}}.$$

Integrating Eq. (34) with respect to *x*_*i*_ we obtain:

35 $$\frac{\partial N}{\partial t}={D}_{E}\frac{{\partial }^{2}N}{\partial {x}_{m}^{2}}-\frac{N}{\tau }+\kappa \hat{T}.$$

Taking in consideration the linearity of the problem and following the same manner Eqs (22) and (23) can be written in the following approximate form:

36 $$\rho \frac{{\partial }^{2}{u}_{i}}{\partial {t}^{2}}=\mu \frac{{\partial }^{2}{u}_{i}}{\partial {x}_{m}^{2}}+(\mu +\lambda )\frac{{\partial }^{2}{u}_{m}}{\partial {x}_{i}^{2}}-\gamma \frac{\partial \hat{T}}{\partial {x}_{i}}-{\delta }_{n}\frac{\partial N}{\partial {x}_{i}}+{\mu }_{0}{H}_{0}^{2}\frac{{\partial }^{2}{u}_{i}}{\partial {x}_{i}^{2}},$$

37 $$\frac{1}{k}\frac{\partial \hat{{\rm{T}}}}{\partial t}=\frac{{\partial }^{2}\hat{\phi }}{\partial {x}_{i}^{2}}-\frac{{E}_{g}}{{K}_{0}\tau }N+\frac{\gamma {T}_{0}}{{K}_{0}}\frac{\partial }{\partial {x}_{i}}\frac{\partial {u}_{i}}{\partial t}.$$

## Mathematical Formulation of the Problem

Considering the effect of the external magnetic field in slowly moving with variable thermal conductivity without body force and no heat source, the one dimension form of Eqs (35), (36), (37) and (30) is:

38 $$\frac{\partial N}{\partial t}={D}_{E}\frac{{\partial }^{2}N}{\partial {x}^{2}}-\frac{N}{\tau }+\kappa \,\hat{T},$$

39 $$\rho \frac{{\partial }^{2}u}{\partial {t}^{2}}=(2\mu +\lambda )(1+{R}_{H})\frac{{\partial }^{2}u}{\partial {x}^{2}}-\hat{\gamma }\frac{\partial \hat{{\rm{T}}}}{\partial x}-{\delta }_{n}\frac{\partial N}{\partial x},$$

40 $$\frac{1}{k}\frac{\partial \hat{{\rm{T}}}}{\partial t}=\frac{{\partial }^{2}\hat{\phi }}{\partial {x}^{2}}-\frac{{E}_{g}}{{K}_{0}\tau }N+\frac{\gamma {T}_{0}}{{K}_{0}}\frac{\partial e}{\partial t},$$

41 $$\hat{T}=\hat{\phi }-a\frac{{\partial }^{2}\hat{\phi }}{\partial {x}^{2}}.$$

Where the magnetic pressure number is ${R}_{H}=\frac{{\mu }_{0}{H}_{0}^{2}}{\hat{\rho }{C}_{T}^{2}}=\frac{{\alpha }_{0}^{2}}{{C}_{T}^{2}}$, which it represent the influence of the magnetic field on plasma-thermal-elastic medium and ${\alpha }_{0}={H}_{0}\sqrt{\frac{{\mu }_{0}}{\rho }}$ is the wave velocity of the semiconductor elastic medium.

For convenience, introduce the dimensionless variables in the above equations as following:

42 $$\begin{array}{c}(x^{\prime} ,u^{\prime} )=\frac{(x,u)}{{C}_{T}{t}^{\ast }},\,t^{\prime} =\frac{t}{{t}^{\ast }},\,T^{\prime} =\frac{\gamma T}{2\mu +\lambda },\\ (\hat{T^{\prime} },\hat{\phi }^{\prime} )=\frac{\gamma (\hat{T},\hat{\phi })}{2\mu +\lambda },\,N^{\prime} =\frac{{\delta }_{n}N}{2\mu +\lambda },\,{\sigma ^{\prime} }_{ij}=\frac{{\sigma }_{ij}}{\mu }.\end{array}$$

Substituting from Eq. (42) into Eqs (38–41), we obtain (the prime is removed for more convenient):

43 $$(\frac{{\partial }^{2}}{\partial {x}^{2}}-{q}_{1}-{q}_{2}\frac{\partial }{\partial t})N+{\varepsilon }_{3}\,\hat{T}=0,$$

44 $$[(1+{R}_{H})\frac{{\partial }^{2}}{\partial {x}^{2}}-\frac{{\partial }^{2}}{\partial {t}^{2}}]u-\frac{\partial \hat{T}}{\partial x}-\frac{\partial N}{\partial x}=0,$$

45 $$\frac{{\partial }^{2}\hat{\phi }}{\partial {x}^{2}}-{q}_{3}\frac{\partial \hat{{\rm{T}}}}{\partial t}+{\varepsilon }_{1}\frac{\partial e}{\partial t}-{\varepsilon }_{2}N=0,$$

46 $$\frac{{\partial }^{2}\hat{\phi }}{\partial {x}^{2}}+\frac{1}{{a}^{\ast }}(\hat{T}-\hat{\phi })=0,$$

47 $${\rm{\sigma }}=\beta (\frac{\partial u}{\partial x}-(T+N)),$$

where,

48 $$\begin{array}{c}{q}_{1}=\frac{k{t}^{\ast }}{{D}_{E}\rho \tau {C}_{e}},\,{q}_{2}=\frac{k}{{D}_{E}\rho {C}_{e}},\,{q}_{3}=\frac{1}{\rho {C}_{e}},\,{\varepsilon }_{1}=\frac{{\gamma }^{2}{T}_{0}{t}^{\ast }}{{K}_{0}\rho },\,{\varepsilon }_{2}=\frac{{\alpha }_{T}{E}_{g}k{t}^{\ast }}{{d}_{n}{K}_{0}\rho \tau {C}_{e}},\\ {t}^{\ast }=\frac{k}{\rho {C}_{e}{C}_{T}^{2}},\,{C}_{T}^{2}=\frac{2\mu +\lambda }{\rho },\,{C}_{L}^{2}=\frac{\mu }{\rho },\,{\beta }^{2}=\frac{{C}_{T}^{2}}{{C}_{L}^{2}},\,{a}^{\ast }=\frac{a}{{C}_{T}^{2}{t}^{\ast 2}},\,\beta =\frac{(2\mu +\lambda )}{\mu }.\end{array}$$

In the expressions (48) *ε*_1_ and *ε*_2_ are the thermoelastic coupling and the thermoenergy coupling parameters respectively.

In order to solve the problem, the initial conditions can be taken as:

49 $$\begin{array}{rcl}{u(x,t)|}_{t=0} & = & {\frac{\partial u(x,t)}{\partial t}|}_{t=0}=0,\\ {\hat{T}(x,t)|}_{t=0} & = & {\frac{\partial \hat{T}(x,t)}{\partial t}|}_{t=0}=0,\\ {\sigma (x,t)|}_{t=0} & = & {\frac{\partial \sigma (x,t)}{\partial t}|}_{t=0}=0,\\ {N(x,t)|}_{t=0} & = & {\frac{\partial N(x,t)}{\partial t}|}_{t=0}=0,\\ {\hat{\phi }(x,t)|}_{t=0} & = & {\frac{\partial \hat{\phi }(x,t)}{\partial t}|}_{t=0}=0.\end{array}$$

## Method of Solution

To obtain the complete solutions of the considered problem, we use the Laplace transform defined for Ψ(*x*, *t*) as:

50 $$L({\rm{\Psi }}(x,t))=\bar{{\rm{\Psi }}}(x,s)={\int }_{0}^{\infty }{\rm{\Psi }}(x,t)\,\exp (\,-\,st)\,dt.$$

Therefore, Eqs (43)–(47) becomes:

51 $$({D}^{2}-\Im )\bar{N}+{\varepsilon }_{3}\,\bar{\hat{T}}=0,$$

52 $$({D}^{2}-{\alpha }_{1})\bar{e}-{\alpha }_{2}{D}^{2}(\bar{\hat{T}}+\bar{N})=0,$$

53 $${D}^{2}\bar{\hat{\phi }}-{q}_{3}s\bar{\hat{T}}+{\varepsilon }_{1}s\bar{e}-{\varepsilon }_{2}\bar{N}=0,$$

54 $$({D}^{2}-\zeta )\bar{\hat{\phi }}+\zeta \bar{\hat{T}}=0,$$

55 $$\bar{{\rm{\sigma }}}=\beta (\bar{e}-(\bar{T}+\bar{N})),$$

where, $$D=\frac{d}{dx}$$, $${D}^{2}=\frac{{d}^{2}}{d{x}^{2}}$$, $$\Im ={q}_{1}+{q}_{2}s$$, $$\zeta =\frac{1}{{a}^{\ast }}$$, $${\alpha }_{1}=\frac{{s}^{2}}{(1+{R}_{H})}$$, $${\alpha }_{2}=\frac{1}{1+{R}_{H}}$$.

To obtain non-trivial solution for the system (51)–(54) we should have:

56 $$|\begin{array}{cccc}\begin{array}{c}0\\ ({D}^{2}-{\alpha }_{1})\\ s{\varepsilon }_{1}\\ 0\end{array} & \begin{array}{c}{\varepsilon }_{3}\\ -{\alpha }_{2}{D}^{2}\\ -s{q}_{3}\\ \zeta \end{array} & \begin{array}{c}({D}^{2}-{\rm{\Im }})\\ -{\alpha }_{2}{D}^{2}\\ -{\varepsilon }_{2}\\ 0\end{array} & \begin{array}{c}0\\ 0\\ {D}^{2}\\ ({D}^{2}-\zeta )\end{array}\end{array}|=0.$$

Using the elimination method technique in the coupled system of Eqs (51)–(54), we obtain the following sixth order differential equation in the variables $\bar{\hat{T}}$, $\bar{e}$, $\bar{N}$ and $\bar{\hat{\phi }}(x)$:

57 $$[{D}^{6}-E{D}^{4}+F{D}^{2}-G]\,\{\bar{e},\bar{\hat{T}},\,\bar{N},\bar{\hat{\phi }}\}(x,s)=0$$

where

58 $${A}_{2}=({\alpha }_{2}{\varepsilon }_{1}-{q}_{3})s-\zeta ,$$

59 $$E={\varepsilon }_{2}{\varepsilon }_{3}-\Im -{\alpha }_{1}+(\Im +\zeta )({\alpha }_{2}{\varepsilon }_{1}-{q}_{3})s+{\alpha }_{2}{\varepsilon }_{1}{\varepsilon }_{3}s)]/{A}_{2},$$

60 $$F=-\,[\Im {\alpha }_{1}-{\varepsilon }_{2}{\varepsilon }_{3}({\alpha }_{1}+\zeta )-\zeta {\alpha }_{2}{\varepsilon }_{1}s(\Im -{\varepsilon }_{3})+(\Im +\zeta ){q}_{3}s)]/{A}_{2},$$

61 $$G=[\zeta {\alpha }_{1}({\varepsilon }_{2}{\varepsilon }_{3}+\Im {q}_{3}s)]/{A}_{2}.$$

Factorizing Eq. (56) as:

62 $$({D}^{2}-{k}_{1}^{2})({D}^{2}-{k}_{2}^{2})\,({D}^{2}-{k}_{3}^{2})\{\bar{e},\bar{\hat{T}},\,\bar{N},\bar{\hat{\phi }}\}(x,s)=0,$$

where, ${k}_{n}^{2}\,(n=1\,,2,3)$ are the roots of the characteristic equation:

63 $${k}^{6}-E{k}^{4}+F{k}^{2}-G=0.$$

Because of linearity, the bounded solutions of Eq. (56) are:

64 $$\{\bar{e},\bar{\hat{T}},\bar{N},\bar{\hat{\phi }}\}=\sum _{i=1}^{3}\{{\bar{e}}_{i},\,{\bar{\hat{T}}}_{i},\,{\bar{N}}_{i},\,{\bar{\hat{\phi }}}_{i}\}.$$

Eq. (63) we can be written as:

65 $$\bar{\hat{T}}={{\rm{\Gamma }}}_{1}{e}^{-{k}_{1}x}+{{\rm{\Gamma }}}_{2}{e}^{-{k}_{2}x}+{{\rm{\Gamma }}}_{3}{e}^{-{k}_{3}x},$$

66 $$\bar{N}={a}_{1}{{\rm{\Gamma }}}_{1}{e}^{-{k}_{1}x}+{a}_{2}{{\rm{\Gamma }}}_{2}{e}^{-{k}_{2}x}+{a}_{3}{{\rm{\Gamma }}}_{3}{e}^{-{k}_{3}x},$$

67 $$\bar{e}={b}_{1}{{\rm{\Gamma }}}_{1}{e}^{-{k}_{1}x}+{b}_{2}{{\rm{\Gamma }}}_{2}{e}^{-{k}_{2}x}+{b}_{3}{{\rm{\Gamma }}}_{3}{e}^{-{k}_{3}x},$$

68 $$\bar{\hat{\phi }}={c}_{1}{{\rm{\Gamma }}}_{1}{e}^{-{k}_{1}x}+{c}_{2}{{\rm{\Gamma }}}_{2}{e}^{-{k}_{2}x}+{c}_{3}{{\rm{\Gamma }}}_{3}{e}^{-{k}_{3}x},$$

where the coupling constants *a*_*i*_, *b*_*i*_ and *c*_*i*_(*i* = 1, 2, 3) are defined as:

69 $${a}_{i}=-\,\frac{{\varepsilon }_{3}}{({k}_{i}^{2}-\Im )},\,i=1,2,3,$$

70 $${b}_{i}=\frac{{\alpha }_{2}{k}_{i}^{2}({k}_{i}^{2}-{\varepsilon }_{3}-\Im )}{({k}_{i}^{2}-{\alpha }_{1})({k}_{i}^{2}-\Im )},\,i=1,2,3,$$

71 $${c}_{i}=-\,\frac{\zeta }{({k}_{i}^{2}-\zeta )},\,i=1,2,3.$$

The displacement distribution can be determined from the equation

72 $$\frac{d\bar{u}}{dx}=\bar{e}(x)={b}_{1}{{\rm{\Gamma }}}_{1}{e}^{-{k}_{1}x}+{b}_{2}{{\rm{\Gamma }}}_{2}{e}^{-{k}_{2}x}+{b}_{3}{{\rm{\Gamma }}}_{3}{e}^{-{k}_{3}x}.$$

Then

73 $$\bar{u}=-\,\sum _{i=1}^{3}\frac{{b}_{i}{{\rm{\Gamma }}}_{i}}{{k}_{i}}\,{e}^{-{k}_{i}x},\,i=1,2,3.$$

Finally, from Eq. (55) we obtain the stress distribution:

74 $$\bar{{\rm{\sigma }}}=\beta \sum _{i=1}^{3}{{\rm{\Gamma }}}_{i}({b}_{i}-{a}_{i}-1){e}^{-{k}_{i}x},\,i=1,2,3.$$

## Applications

Since the medium is homogeneous and initially at rest loaded by mechanical forces (ramp type load force) on its surface. To determine the value of the parameters Γ_*i*_, *i* = 1, 2, 3, we consider the following boundary conditions at *x* = 0:

(I) The thermal boundary in non-dimensional, as thermal shock boundary, form is:
  75 $$\phi (0,t)={\phi }_{1}H(t)$$
  Eq. (74) in Laplace transform domain take the form
  76 $$\bar{\phi }(0,s)=\frac{{\phi }_{1}}{s}.$$
  Using Eq. (17), we get
  77 $$\bar{\hat{\phi }}(0,s)=\frac{{\rm{\Lambda }}}{s},$$
  where, $${\rm{\Lambda }}={\phi }_{1}+\frac{{K}_{1}}{2}{\phi }_{1}^{2}$$, hence
  78 $$\sum _{i=1}^{3}{c}_{i}{{\rm{\Gamma }}}_{i}{e}^{-{k}_{i}x}=\frac{{\rm{\Lambda }}}{s}.$$
(II) The normal stress subjected to ramp type load:
  79 $$\sigma (x,t)=\{\begin{array}{ll}0 & t\le 0\\ \frac{t}{{t}_{0}} & 0 < t\le {t}_{0}\\ 1 & t > {t}_{0}\end{array}.$$
  Applying Laplace transform on both sides of (78) we obtain:
  80 $$\bar{\sigma }(0,s)=\frac{(1-{e}^{-s{t}_{0}})}{{t}_{0}{s}^{2}}.$$
  Therefor
  81 $$\sum _{i=1}^{3}{{\rm{\Gamma }}}_{i}({b}_{i}-{a}_{i}-1)=\frac{(1-{e}^{-s{t}_{0}})}{\beta {t}_{0}{s}^{2}},\,i=1,2,3.$$
(III) The transport and recombination processes of the photogenerated carrier boundary condition in Laplace domain transform is:

82 $$\bar{N}(0,\,s)=\frac{{{{-}\kern-0.5em {\lambda}}}}{{D}_{e}}\bar{R}(s),$$

where *R*(*s*), *H*(*t*) are the Heaviside unit step function and ${\rm{{-}\kern-0.5em {\lambda}}}$ is an arbitrary constant. Hence Eq. (81) take the following form:

83 $${a}_{1}{{\rm{\Gamma }}}_{1}+{a}_{2}{{\rm{\Gamma }}}_{2}+{a}_{3}{{\rm{\Gamma }}}_{3}=\frac{{\rm{{-}\kern-0.5em {\lambda}}}}{s{\varepsilon }_{3}{D}_{e}}.$$

The unknown variables Γ_*i*_ can be obtained from Eqs (77), (80) and (82).

Using the mapping Eqs (16) and (17), and the liner form of the thermo-dynamical temperature (13) with the heat conduction (15) equations, we obtain

84 $$\hat{T}=\frac{1}{{K}_{0}}{\int }_{0}^{T}{K}_{0}(1+{K}_{1}T)dT=T+\frac{{K}_{1}}{2}{T}^{2}=\frac{{K}_{1}}{2}{(T+\frac{1}{{K}_{1}})}^{2}-\frac{1}{2{K}_{1}},$$

or

85 $$T=\frac{1}{{K}_{1}}[\sqrt{1+2{K}_{1}\hat{T}}-1].$$

In Laplace transform domain Eq. (84) takes the form:

86 $$\bar{T}=\frac{1}{{K}_{1}}[\sqrt{1+2{K}_{1}\bar{\hat{T}}}-1],$$

and the heat conduction temperature takes the form:

87 $$\bar{\phi }=\frac{1}{{K}_{1}}[\sqrt{1+2{K}_{1}\hat{\phi }}-1].$$

## Numerical Inversion of the Laplace Transform

To obtain the non-dimensional numerical results of the thermo-dynamical temperature, heat conduction temperature, displacement, strain, stress, and carrier density in the time domain, we use the inverse Laplace transformation according to Riemann-sum approximation method.

Any function *f*(*x*, *t*′) can be inverted in Laplace domain, as

88 $$f(y,\,t^{\prime} )={L}^{-1}\{\bar{f}(y,s)\}=\frac{1}{2\pi i}{\int }_{n-i\infty }^{n+i\infty }\exp (st^{\prime} )\bar{f}(y,\,s)ds.$$

Introducing *s* = *n* + *i*Μ(*n*, Μ ∈ *R*), in Eq. (87) we obtain:

89 $$f(y,\,t^{\prime} )=\frac{\exp (nt^{\prime} )}{2\pi }{\int }_{\infty }^{\infty }\exp (i\beta t)\bar{f}(y,n+i\beta )d\beta .$$

Using Fourier series expansion, the function *f*(*y*, *t*′) can be expanded in the interval [0, 2*t*′] and take the following form:

90 $$f(y,\,t^{\prime} )=\frac{{e}^{nt^{\prime} }}{t^{\prime} }[\frac{1}{2}\bar{f}(y,\,n)+{Re}\sum _{k=1}^{N}\bar{f}(y,\,n+\frac{ik\pi }{t^{\prime} })\,{(-1)}^{n}],$$

where *Re* represent the real part, $i=\sqrt{-1}$, provided that *nt*′ ≈ 4.7.

## Numerical Results and Discussions

For numerical evaluations we chose silicon (Si) as semiconductor elastic crystal material. The constants (parameters in SI unit) of the problem as silicon are listed in Table 1^20,34^:

**Table 1:** The physical constants of silicon semiconductor materiel.

*λ* = 3.64 × 10^10^ *N*/*m*^2^	*μ* = 5.46 × 10^10^ *N*/*m*^2^	*ρ* = 2330 *kg*/*m*^3^	*τ* = 5 × 10^−5^ *s*
*T*_0_ = 800 K	*d*_*n*_ = −9 × 10^−31^ *m*^3^	*DE* = 2.5 × 10^−3^ *m*^2^/*s*	*Eg* = 1.11 *eV*
*C*_*e*_ = 695 *J*/(*kg K*)	*α*_*T*_ = 3 × 10^−6^ *K*^−1^	*s* = 2 *m*/*s*	*t* = 8 × 10^−8^ *s*
*μ*_0_ = 4*π* × 10^−7^ *H*/*m*	*K*_0_ = 386 *N*/*K* sec	*λ* = 2 *m*/*s*	*q* = 10^20^ *m*^−3^

Because of the short time duration and non-dimensional forms of the problem, the imaginary parts of the physical quantities distributions are neglected.

### The influence of the two-temperature parameter

**Figure 1:**
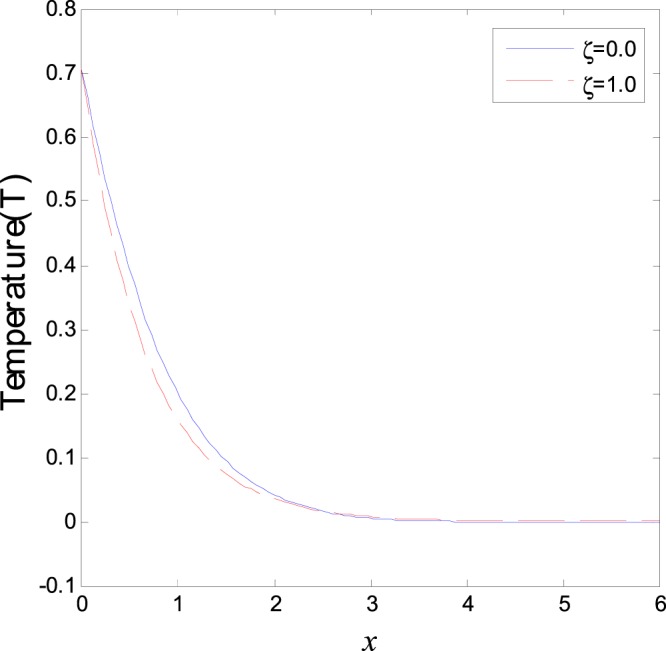
Variation of temperature distribution with different values of ζ in magnetic field at *K*_1_ = −0.1.

**Figure 2:**
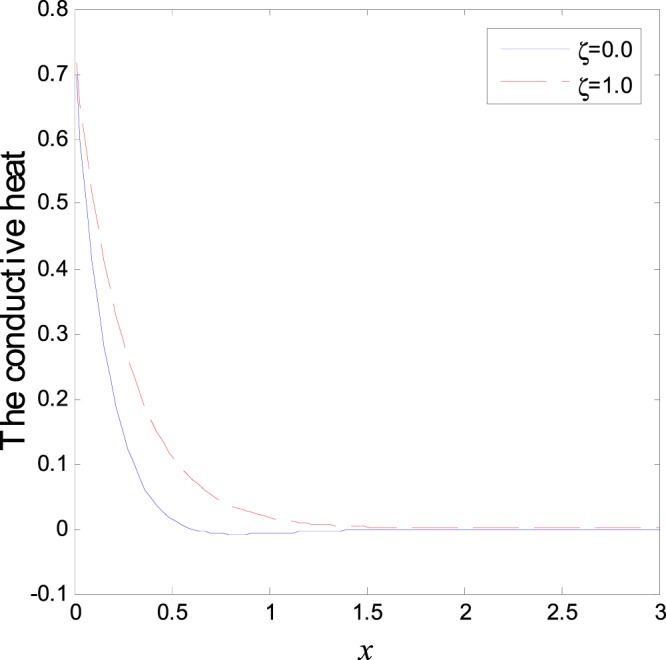
Variation of conductive heat distribution with different values of ζ in magnetic field at *K*_1_ = −0.1.

**Figure 3:**
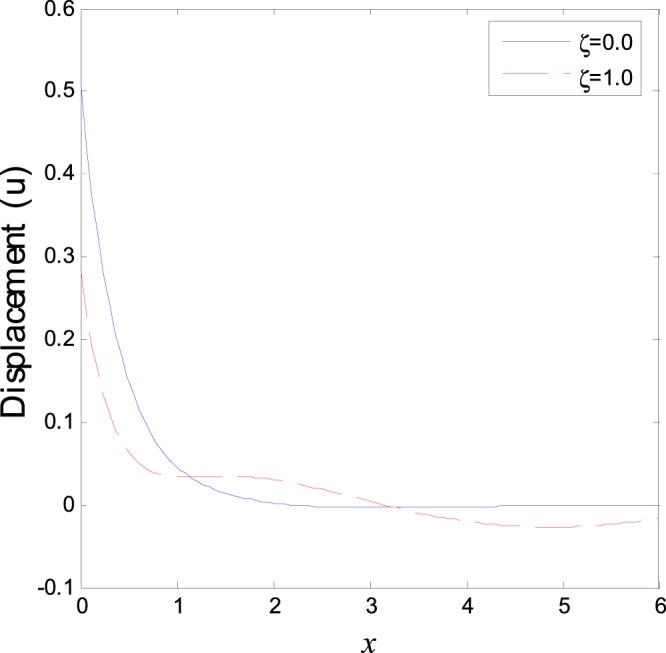
Variation of displacement distribution with different values of ζ in magnetic field at *K*_1_ = −0.1.

**Figure 4:**
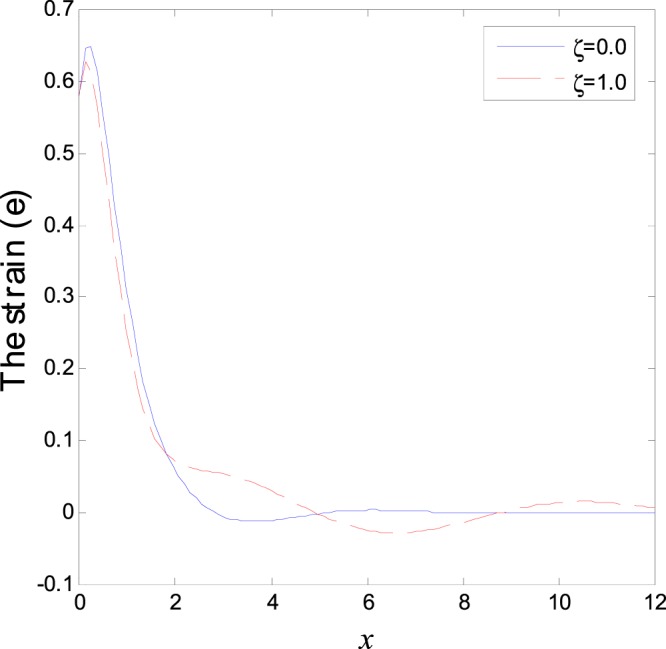
Variation of strain distribution with different values of ζ in magnetic field at *K*_1_ = −0.1.

**Figure 5:**
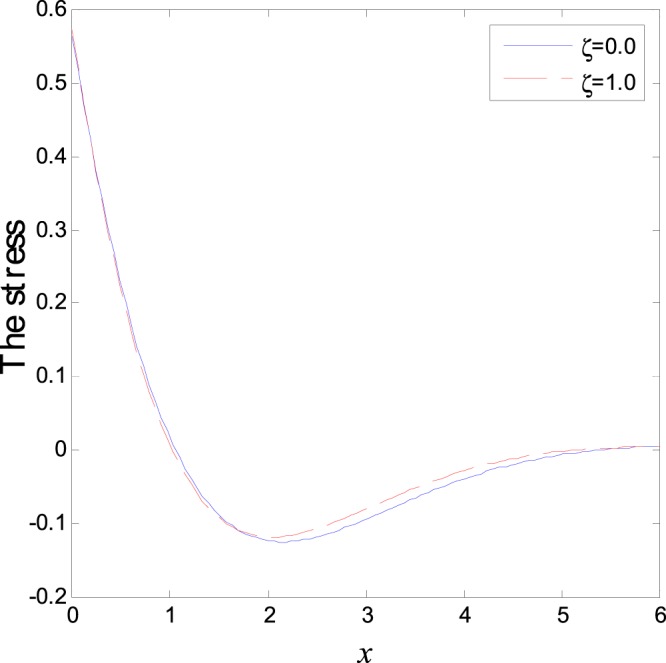
Variation of normal stress distribution with different values of ζ in magnetic field at *K*_1_ = −0.1.

**Figure 6:**
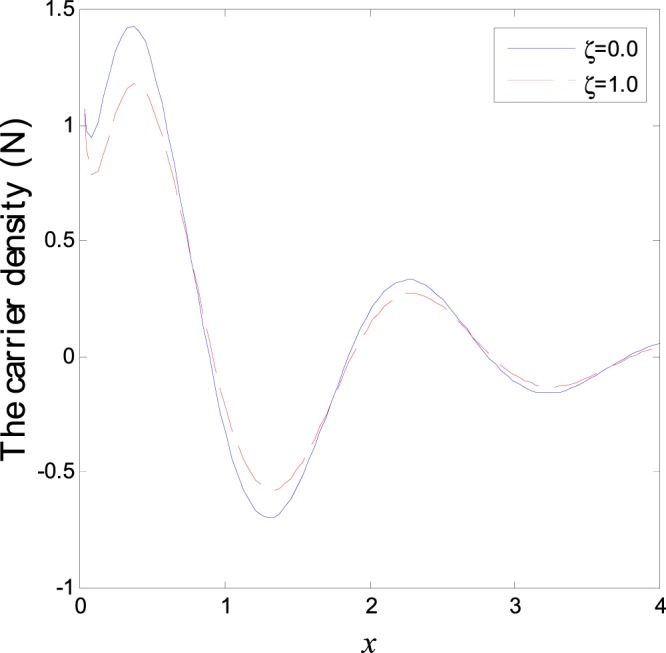
Variation of carrier density distribution with different values of ζ in magnetic field at *K*_1_ = −0.1.

In the first category of Figs (1–6), we established the effect of the two-temperature parameters, *ζ* = {0, 1}, on the behavior of the thermodynamical and conductive temperatures, displacement, strain, stress and the carrier density with the distance *x* (the initial boundary conditions are same for all physical quantitates), the results are shown graphically in Figs (1–6). This study is considered in the presence of the magnetic field and the thermal conductivity (*K*_1_ = −0.1). Also *ζ* = 0 represents the one temperature case while *ζ* = 1 represents the two temperature one. Figure 1 shows the effect of photo-thermal beam light on temperature distribution, the results show that the temperature gets its maximum value near the surface, then begins to decrease gradually and vanishes away of the surface. The same behavior is noticed for the conductive heat distribution as shown in Fig. 2. This behavior agrees with the exponential form of the wave propagation. Also, as shown in Fig. 3, the displacement distribution, when *ζ* = 0 and *ζ* = 1, approximately behave the same. As shown in Fig. 4, due to the thermal pressure the strain tensor starts from positive value at the beginning and increases until it reach the maximum value near the surface. As the distance increases, the strain decreases gradually to reach its minimum value.

The normal stress force starts from a positive value near the surface and then begins to decrease gradually until it reaches the minimum value away from the surface as shown in Fig. 5. Due to the photons stress effect, the carrier density behavior (free charges distribution) starts from positive value and decreases sharply. Afterward it increases gradually to reach the maximum value in the first interval near the surface while in the second interval it decreases smoothly to arrive the minimum value as it moves far from the surface as shown Fig. 6. As the distance increase, same behavior is repeated periodically until it approaches the zero value. This behavior characterizes the semiconductors materials. The difference between the two temperatures theory and one temperature theory is that this theorem differentiates between the wave propagation of the temperature that comes from the thermal process (heat conduction) and that which comes from the mechanical process (thermodynamics temperature).

### The influence of the variable thermal conductivity

**Figure 7:**
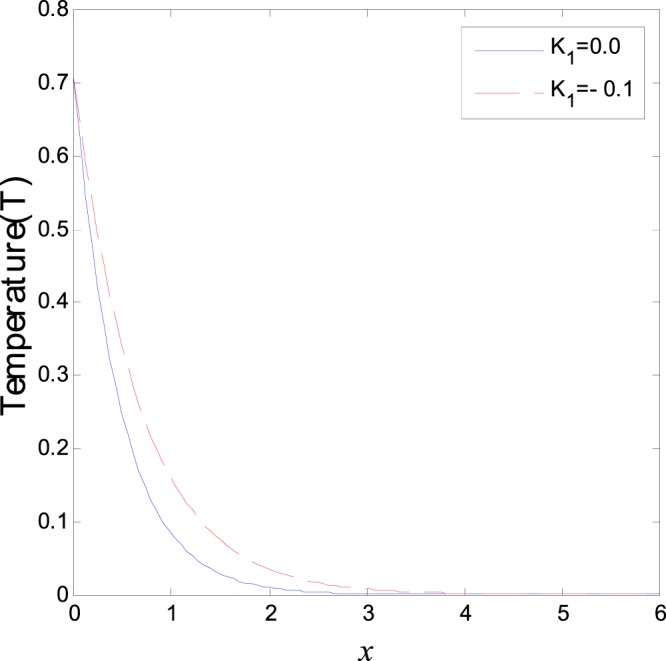
Variation of temperature distribution with different values of *K*_1_ in magnetic field at *ζ* = 1.

**Figure 8:**
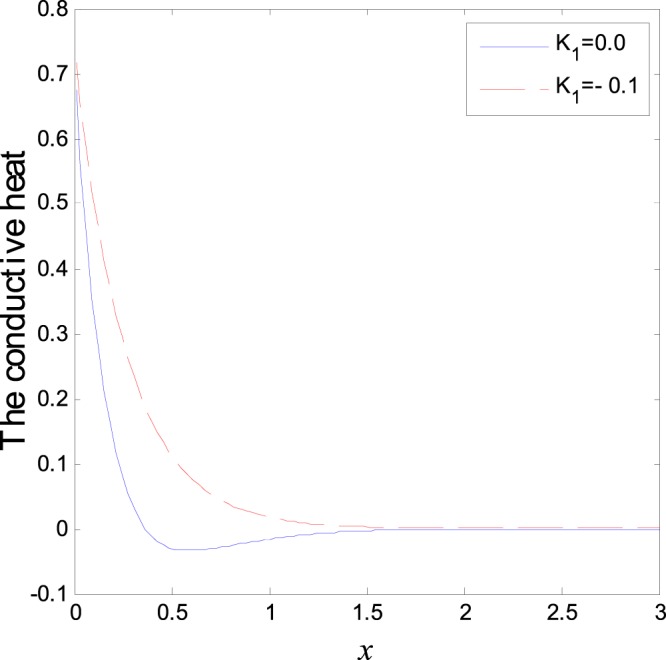
Variation of conductive heat distribution with different values of *K*_1_ in magnetic field at *ζ* = 1.

**Figure 9:**
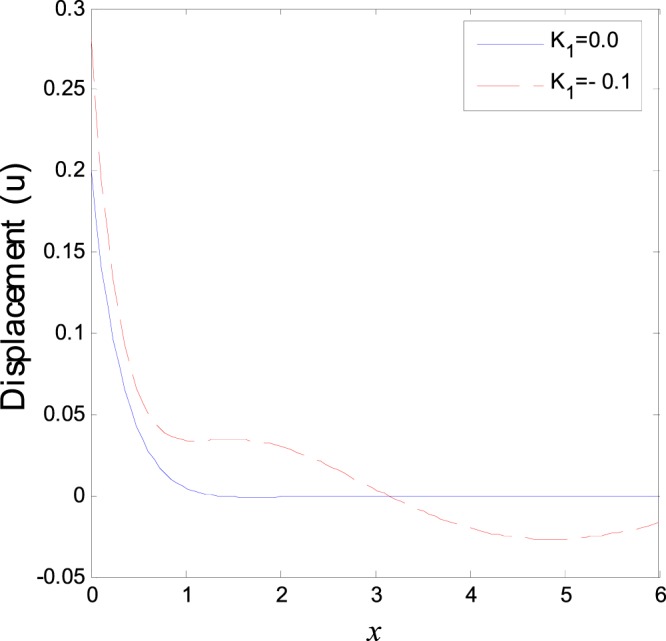
Variation of displacement distribution with different values of *K*_1_ in magnetic field at *ζ* = 1.

**Figure 10:**
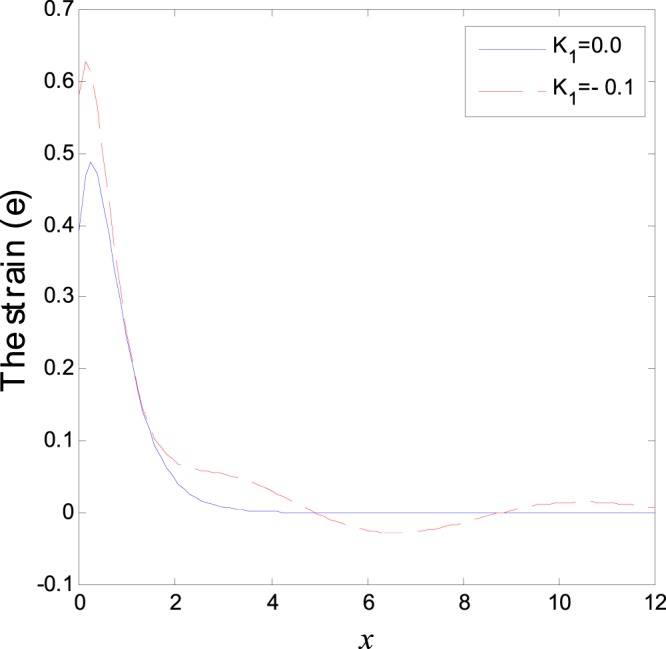
Variation of strain distribution with different values of *K*_1_ in magnetic field at *ζ* = 1.

**Figure 11:**
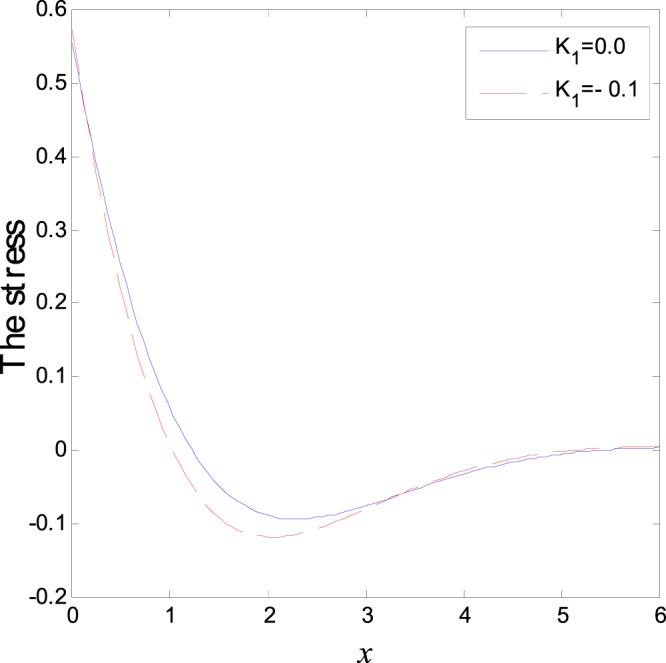
Variation of normal stress distribution with different values of *K*_1_ in magnetic field at *ζ* = 1.

**Figure 12:**
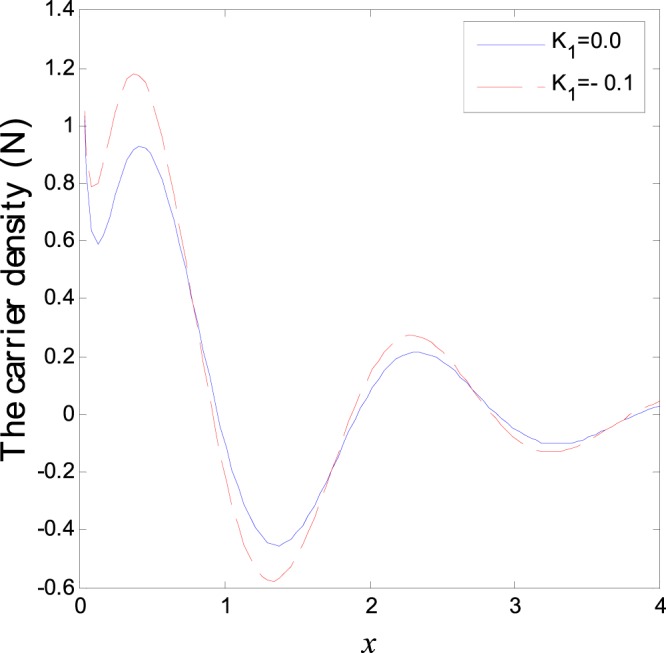
Variation of carrier density distribution with different values of *K*_1_ in magnetic field at *ζ* = 1.

The second category of Figs (7–12), shows the variations of the thermodynamical temperature, the conductive temperature, displacement, strain, stress and carrier density, respectively, as functions of *x*, for different values of the thermal conductivity. All the calculations are obtained presence of both the two-temperature and the magnetic field. The classical case (*K*_1_ = 0.0) is defined when the thermal conductivity is independent of the temperature. While the non-classical case (*K*_1_ = −0.1) can be obtained when the thermal conductivity depends on the temperature. It is clear that the boundary conditions are satisfied for all fields. Also the variations of all fields, approximately, behave the same with respect to the variation in the thermal conductivity magnitude. It is observed that any small changes in the thermal conductivity lead to a significant change in the propagation of wave behavior. Moreover the characteristic curves of the physical quantities start from maximum values then begin to intersect and coincide, finally approach zero value for large distance *x*, which conform the physical equilibrium of the semiconductors elastic solid.

### Effect of the magnetic field

**Figure 13:**
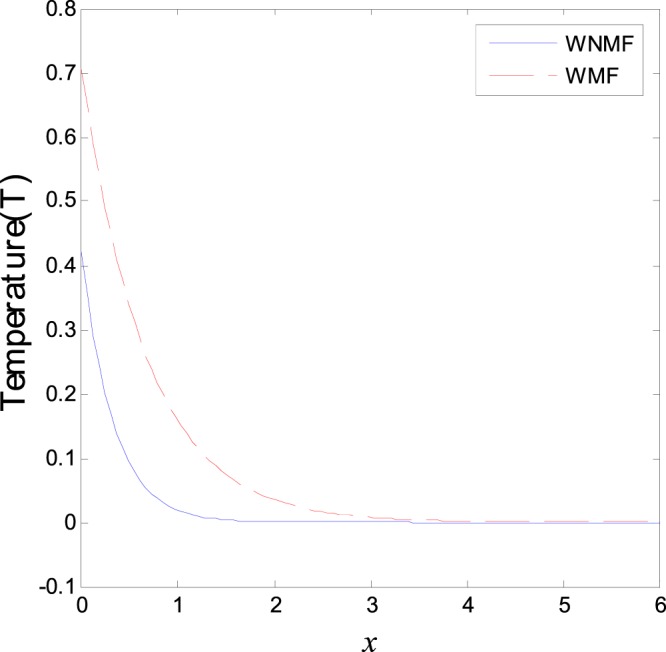
Variation of temperature distribution with different values of magnetic field at *ζ* = 1 and *K*_1_ = −0.1.

**Figure 14:**
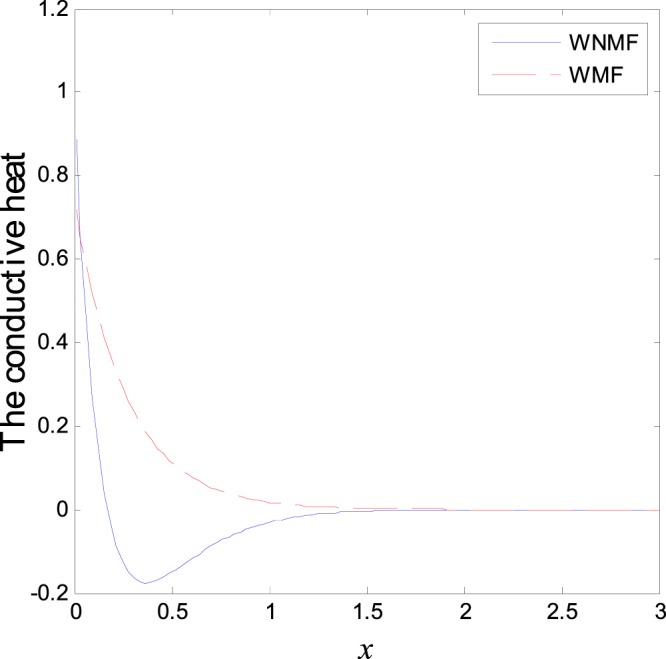
Variation of conductive heat distribution with different values of magnetic field at *ζ* = 1 and *K*_1_ = −0.1.

**Figure 15:**
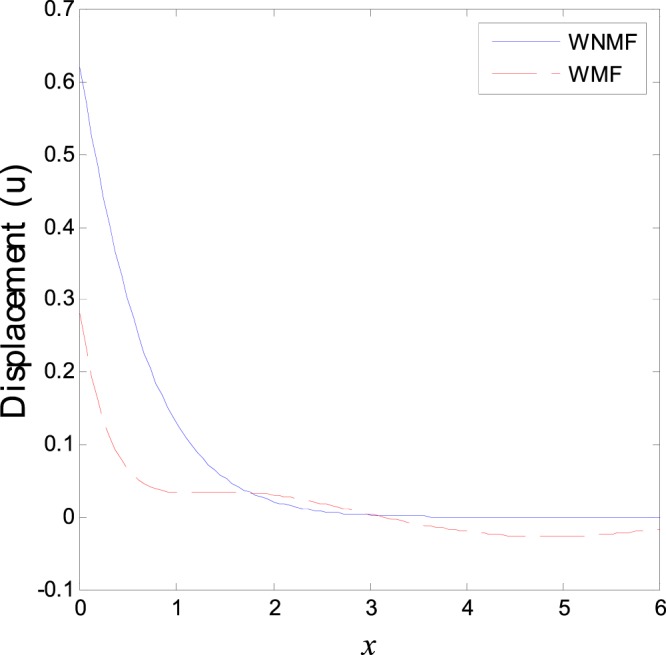
Variation of displacement distribution with different values of magnetic field at *ζ* = 1 and *K*_1_ = −0.1.

**Figure 16:**
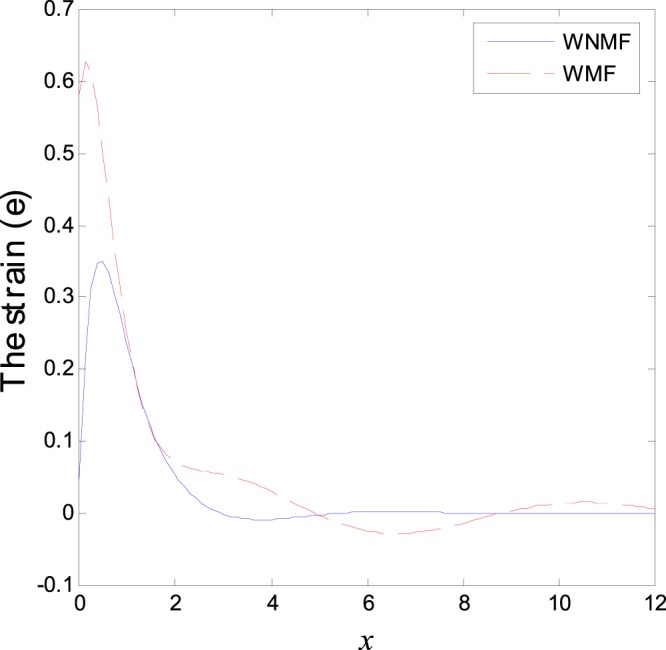
Variation of strain distribution with different values of magnetic field at *ζ* = 1 and *K*_1_ = −0.1.

**Figure 17:**
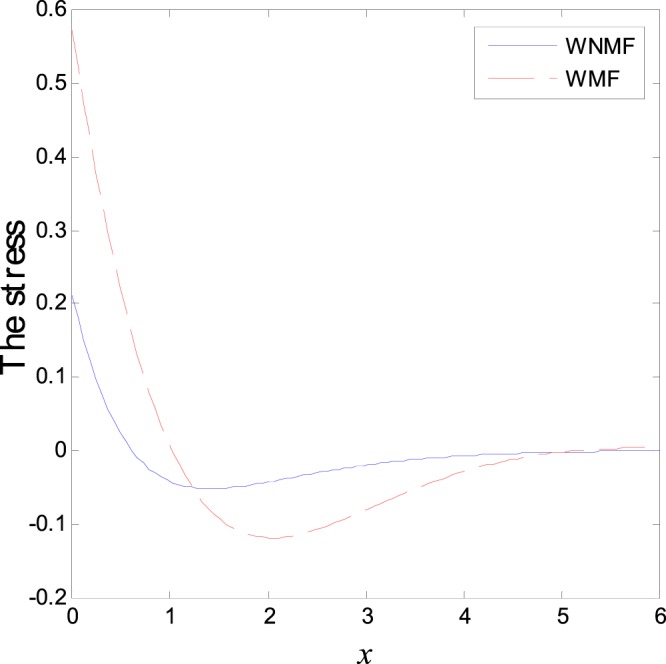
Variation of normal stress distribution with different values of magnetic field at *ζ* = 1 and *K*_1_ = −0.1.

**Figure 18:**
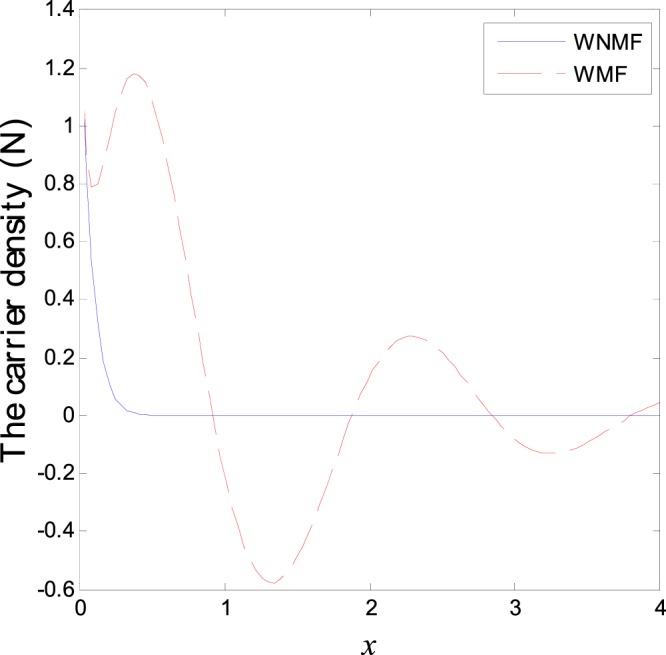
Variation of carrier density distribution with different values of magnetic field at *ζ* = 1 and *K*_1_ = −0.1.

The third category of Figs (13–18) explains the effect of the initial magnetic field *H*_0_ on the physical fields with respect to the *x* in two cases without magnetic field WNMF (solid line) and with magnetic field WMF (dashed line). The calculations are carried out for *K*_1_ = −0.1 and *ζ* = 1. In this case, the behavior of the physical fields is deferent because the influence of the initial magnetic field rearranges the particles in the elastic medium. This explains why the curves in the figures are compact to each other as *x* tends to infinity. These results satisfy the physical fact for the behavior of the semiconductors to reach its equilibrium state of particles.

## Conclusion

During this study the effect of two-temperature parameter, the variable thermal conductivity, and magnetic field in context of the Photothermal and thermoelasticity theories is considered. We concluded that: the two-temperature case gave more accurate results than the one-temperature case. The two- temperature parameter has great influence on all physical fields of the problem under investigation. It is necessary to separate between the thermodynamic temperature and the heat conductive. Some physical fields depend on thermal conductivity, the thermal conductivity affects on the thermo-mechanical behavior of semiconductors. The variable thermal conductivity plays a great rule in our study, which has a significant effect on all fields. The magnetic field has a good effect on all the physical fields. It is observed that the carrier density has a deferent oscillatory structure from other physical quantities.

## References

[1] Hasselman D, Heller R (1980). Thermal Stresses in Severe Environments.

[2] Youssef H (2005). State-Space on Generalized Thermoelasticity for an Infinite Material with a Spherical Cavity and Variable Thermal Conductivity Subjected to Ramp-Type Heating. Journal of CAMQ, Applied Mathematics Institute.

[3] Youssef, H. & El-Bary, A. Thermal Shock Problem of a Generalized Thermoelastic Layered Composite Material with Variable Thermal Conductivity, *Math. Prob. Eng*. Article ID 87940, 1–14 (2006).

[4] Youssef H, Abbas I (2007). Thermal shock problem of generalized thermoelasticity for an infinite long annular cylinder with variable thermal conductivity. Comp. Methods in Science and Technology..

[5] Todorović DM, Nikolić PM, Bojičić AI (1999). Photoacoustic frequency transmission technique: electronic deformation mechanism in semiconductors. J. Appl. Phys..

[6] Gordon JP (1965). Long-transient effects in lasers with inserted liquid samples. J App.l Phys..

[7] Kreuzer LB (1971). Ultralow gas concentration infrared absorption spectroscopy. J Appl. Phys..

[8] Tam, A. C. *Ultrasensitive laser spectroscopy*. 1–108 (New York (NY): Academic Press, 1983).

[9] Tam AC (1986). Applications of photoacoustic sensing techniques. Rev. Mod. Phys..

[10] Tam, A. C. *Photothermal investigations in solids and fluids*. 1–33 (Boston (MA): Academic Press, 1989).

[11] Rosencwaig A, Opsal J, Smith WL, Willenborg DL (1985). Detection of thermal waves through optical reflectance. Appl. Phys. Lett..

[12] Opsal J, Rosencwaig A (1982). Thermal wave depth profiling: Theory. J Appl. Phys..

[13] Song YQ (2010). Study on the generalized thermoelastic vibration of the optically excited semiconducting microcantilevers. Int. J. Solids Stru..

[14] Lotfy K (2016). The elastic wave motions for a photothermal medium of a dual-phase-lag model with an internal heat source and gravitational field. Can J. Phys..

[15] Lotfy KA (2018). Novel Model of Photothermal Diffusion (PTD) fo Polymer Nano- composite Semiconducting of Thin Circular Plate. Physica B- Condenced Matter.

[16] Lotfy K, Kumar R, Hassan W, Gabr M (2018). Thermomagnetic effect with microtemperature in a semiconducting Photothermal excitation medium. Appl. Math. Mech. Eng. Ed..

[17] Lotfy K, Gabr M (2017). Response of a semiconducting infinite medium under two temperature theory with photothermal excitation due to laser pulses. Optics and Laser Tech..

[18] Lotfy K (2017). Photothermal waves for two temperature with a semiconducting medium under using a dual-phase-lag model and hydrostatic initial stress. Waves in Random and Complex Media..

[19] Lotfy K, Hassan W, Gabr M (2017). Thermomagnetic effect with two temperature theory for Photothermal process under hydrostatic initial stress. Results in Phys..

[20] Hobiny A, Abbas I (2016). A study on photothermal waves in an unbounded semiconductor medium with cylindrical cavity. Mech Time-Depend Mater..

[21] Hobiny A, Abbas I (2018). Analytical solutions of photo-thermo-elastic waves in a non-homogenous semiconducting material. Results in Physics..

[22] Abo-dahab S, Lotfy K (2017). Two-temperature plane strain problem in a semiconducting medium under photothermal theory. Waves in Random and Complex Media..

[23] Chen PJ, Gurtin ME (1968). On a theory of heat conduction involving two temperatures. Z. Angew. Math. Phys..

[24] Chen PJ, Gurtin ME, Williams WO (1968). A note on non-simple heat conduction. Z. Angew. Math. Phys..

[25] Chen PJ, Gurtin ME, Williams WO (1969). On the thermodynamics of non-simple elastic materials with two temperatures. Z. Angew. Math. Phys..

[26] Youssef HM (2006). Theory of two-temperature-generalized thermoelasticity. IMA Journal of Applied Mathematics.

[27] Mandelis A, Nestoros M, Christofides C (1997). Thermoelectronic-wave coupling in laser photothersmal theory of semiconductors at elevated temperature. Opt. Eng..

[28] Todorovic DM (2003). Plasma, thermal, and elastic waves in semiconductors. Rev. Sci. Instrum..

[29] Vasil’ev AN, Sandomirskii VB (1984). Photoacoustic effects in finite Semiconductors. Sov. Phys. Semicond..

[30] Christofides C, Othonos A, Loizidou E (2002). Influence of temperature and modulation frequency on the thermal activation coupling term in laser Photothermal theory. J. Appl. Phys..

[31] Tritt, T. M. *Thermal Conductivity Theory, Properties, and Applications*. (Springer, 2004).

[32] Vandersande J, Wood C (1986). The thermal conductivity of insulators and Semiconductors. Contemporary Physics..

[33] Youssef H, El-Bary A (2010). Two-Temperature Generalized Thermoelasticity with Variable Thermal Conductivity. Journal of Thermal Stresses..

[34] Song YQ, Bai JT, Ren ZY (2012). Study on the reflection of photothermal waves in a semiconducting medium under generalized thermoelastic theory. Acta Mech..

